# Disputed paternity presumption in Burkina Faso: determination of the biological fathers of children using ABO-rhesus/hemoglobin electrophoresis and STR assays

**DOI:** 10.1186/s43141-021-00221-3

**Published:** 2021-08-30

**Authors:** Missa Millogo, Serge Theophile Soubeiga, Bapio Valerie Jean Telesphore Bazie, Theodora Mahoukede Zohoncon, Abdoul Karim Ouattara, Albert Theophane Yonli, Jacques Simpore

**Affiliations:** 1 Direction of Scientific and Technical Police, Ouagadougou, Burkina Faso; 2 Laboratory of Molecular Biology and Genetics (LABIOGENE) of University Joseph Ki-Zerbo, P.O. 03 BOX, 7021 Ouaga 03, Ouagadougou, Burkina Faso; 3 Biomolecular Research Centre Pietro Annigoni (CERBA)), P.O. 01 BOX 364, Ouagadougou, Ouaga 01 Burkina Faso; 4 Research Institute of Health Sciences (IRSS)), P.O. 03. BOX 7192, Ouagadougou, Ouaga 03 Burkina Faso; 5 Research Institute of Applied and Technical Sciences (IRSAT)), P.O. 03 BOX 7047, Ouagadougou, Ouaga 03 Burkina Faso; 6 University of Saint Thomas d’Aquin (USTA)), P.O. 06 BOX 10212, Ouagadougou, Ouaga 06 Burkina Faso

**Keywords:** Paternity, ABO-rhesus, Hemoglobin electrophoresis of Short Tandem Repeat, Burkina Faso, West Africa

## Abstract

**Background:**

In resource-limited countries, ABO, HLA, MNS, Kells, and hemoglobin electrophoresis are classic tests for the resolution of paternity disputes due to their affordable cost. The limitations of these tests in cases of disputed paternity require the use of Short Tandem Repeats (STR) for their certification. This study aimed to determine the biological fathers of children using ABO-rhesus/hemoglobin electrophoresis and STR assays in Burkina Faso, West Africa.

**Results:**

Of the fourteen trios studied, the ABO-rhesus/hemoglobin electrophoresis analysis revealed ten probable inclusion cases, three exclusion cases, and one undetermined paternity. DNA STR analysis found five inclusions of paternity out of the ten probable inclusions with ABO-rhesus/hemoglobin electrophoresis assay versus nine exclusions of paternity.

**Conclusion:**

This study showed that the implementation of the analysis of short tandem repeat is required to resolve increasing disputed filiation cases in Burkina Faso.

## Background

The biological determination of filiations is an old problem. The analysis is based on the genetic polymorphisms of individuals and their Mendelian transmission [1, 2]. Referring to techniques based on blood group determination, some authors showed that in affiliation cases, a combined exclusion chance for non-fathers of 99.995% is obtained by the examination of well-established blood group systems [3]. The issues in the use of these methods to include paternity of “alleged fathers” are related to the random transmission of alleles in the general population. The late maturation of antigens is a barrier to the determination of filiation using the ABO system. Thus, the establishment of parentage, by the current tests such as ABO, HLA, MNS, and Kell, is not accurate, with low exclusion probabilities around 0.17 [4]. Moreover, hemoglobin (Hb) electrophoresis can be used for paternity dispute cases based on the allelic variants between individuals [5, 6]. However, the technique is limited when there is correspondence between the Hb genotype of the alleged father–mother and child. Taking these limitations into account, in the context of paternity research, it is necessary to combine several systems [ABO, rhesus, HLA, MNS, Kell, serum systems...] or to use other more efficient systems such as microsatellite genetic analysis, or “short tandem repeats” (STR) [7, 8]. Hemoglobin electrophoresis is complementary to ABO-rhesus because the first is for “exclusion” when the latter detected “inclusion”.

STR is a polymorphic locus present in all eukaryotic genomes. They generally consisted of tandem matrices of short repeated sequences of 2 to 6 base pairs, and polymorphism occurs when the number of copies of the repeated sequence present at a given STR locus varies between individual chromosomes [9–11]. Hundreds of microsatellites have been studied and some are used as markers for the determination of genetic fingerprints to discriminate or genetically link individuals (families, immigrants, etc.) [12]. They present a wide diversity and can be used in the identification of paternity testing cases [8, 13]. The application of STRs to the search for parentage in 877 paternity cases had in the past ruled out 35.2% of cases and found a probability of paternity of 99.9999% [14]. In Burkina Faso, the justice system is facing strong demands for paternity tests, causes of divorce, and family conflicts. Despite the technical limitations with a high risk of misidentification of the biological father, ABO-rhesus/Hb electrophoresis has been used for a long time to resolve paternity disputed cases in Burkina Faso, because of their affordability and the absence of STR assays. For a deep identification, the present pioneer study aimed to determine the biological fathers of children using the old ABO-rhesus/Hb electrophoresis method and STR assays for the first time in Burkina Faso, West Africa.

## Methods

### Sample collection

Fourteen trios (mother-child-alleged father) were involved in the present study. They were referred to the Pietro Annigoni Biomolecular Research Centre for samples (42) collection and paternity tests at the request of the Tribunal de Grande Instance de Ouagadougou. Written informed consent was obtained from participants before blood sample collection on EDTA tube for blood grouping and Hb electrophoresis and on FTA paper (NucleiCard, Brescia, Italy).

### Carrying out blood grouping and hemoglobin electrophoresis

The determination of the blood and rhesus groups was performed using the Beth-Vincent technique with Anti-A, Anti-B, Anti-AB, and Anti-D sera. The determination of the hemoglobin type was performed using the HELENA electrophoresis chain (Helena Biosciences Europe, Queensway South, Gateshead Tyne, and Wea) according to the manufacturer’s instructions. Hemolysate was prepared by mixing 1 V of the whole blood with 3 V of Helena hemolysis reagent (0.005 M EDTA and 0.01% potassium cyanide). Electrophoresis was performed at 350 V for 25 min in a boric acid/Tris-EDTA buffer (pH 8.4, ionic strength = 0.035).

### Amplification by polymerase chain reaction (PCR)

PCR amplification was performed using 1.2 mm of bloodstained disc obtained by a punch on FTA paper previously soaked in blood and containing 5 to 20 ng of DNA. A multiplex PCR amplification of 16 loci of tandem repeat strap (polymorphic STR loci) was performed using the AmpFlSTR® identifiler® Direct kit (Applied Biosystems, Foster City, CA, USA) according to the manufacturer’s instructions. Among the 16 STRs, the Amelogenin marker was included to allow genetic identification of the sex of each subject. The characteristics of the 16 STRs are shown in Table 1. The PCR was performed in 25 μL of reaction volume containing 5–20 ng DNA, 12.5 μL primers, and 12.5 μL Master Mix on the Gene Amp PCR System 9700 thermocycler (Applied Biosystems, USA) according to the following program: initial denaturation at 94°C for 11 min, 28 cycles of 9°C for 20 s, 59°C for 3 min, and 72°C for 1 min, and final extension at 60°C for 25 min.

**Table 1:** 16 STR loci and alleles with their characteristics

Locus	Location on the chromosome	Included alleles	Fluorochrome
D8S1179	8	8, 9 10, 11, 12, 13, 14, 15, 16, 17, 18, 19	6-FAM
D21S11	21q11.2-q21	24, 24.2, 25, 26, 27, 28, 28.2, 29, 29.2, 30, 30.2, 31, 31.2, 32, 32.2, 33, 33.2, 34, 34.2, 35, 35.2, 36, 37, 38	
D7S820	7q11.21-22	6, 7, 8, 9, 10, 11, 12, 13, 14, 15	
CSF1PO	5q33.3-34	6, 7, 8, 9, 10, 11, 12, 13, 14, 15	
D3S1358	3p	12, 13, 14, 15, 16, 17, 18, 19	VIC
TH01	11p15.5	4, 5, 6, 7, 8, 9, 9.3, 10, 11, 13.3	
D13S317	13q22-31	8, 9, 10, 11, 12, 13, 14, 15	
D16S539	16q24-qter	5, 8, 9, 10, 11, 12,13, 14, 15	
D2S1338	2q35-37.1	15, 16, 17, 18, 19, 20, 21, 22, 23, 24, 25, 26, 27, 28	
D19S433	19q12-13.1	9, 10, 11, 12, 12.2, 13, 13.2, 14, 14.2, 15, 15.2, 16, 16.2, 17, 17.2	NED
vWA	12p12-pter	11,12, 13, 14, 15, 16, 17, 18, 19, 20, 21, 22, 23, 24	
TPOX	2p23-2per	6, 7, 8, 9, 10, 11, 12, 13	
D18S51	18q21.3	7, 9, 10, 10.2, 11, 12, 13, 13.2, 14, 14.2, 15, 16, 17, 18, 19, 20, 21, 22, 23, 24, 25, 26, 27	
Amelogenin	X: p22.1-22.3 Y: p11.2	X, Y	PET
D5S818	5q21-31	7, 8, 9, 10, 11, 12, 13, 14, 15, 16	
FGA	4q28	17, 18, 19, 20, 21, 22, 23, 24, 25, 26, 26.2, 27, 28, 29, 30, 30.2, 31.2, 32.2, 33.2, 42.2, 43.2, 44.2, 45.2, 46.2, 47.2, 48.2, 50.2, 51.2	

### Capillary electrophoresis

The amplification fragments obtained were then analyzed on the ABI 3130 Genetic Analyzer (Applied Biosystem, USA) on a 96-well plate containing 1 μL of PCR product, 8.7 μL of Hi-Di Formamide, and 0.3 μL of GeneScan 500 LIZ Size Standard followed by denaturation at 95°C for 3 min and immediate cooling on ice for 3 min. The electrophoresis was performed with Performance-Optimized Polymer 4 (POP4) with a capillary of 36 cm. After electrophoresis, GeneMapper® ID version v3.2.1 software was used to assemble the obtained sequences and compares them to the allele scale to determine the allele types present in each analyzed sample.

### Statistical analyses

The paternity index (PI), which measures the weight of scientific evidence obtained from the paternity test, was calculated for each STR locus using the method described by Eisenberg, 2003. Then, the combined paternity index (CPI) was estimated by multiplying the individual paternity index with the others. The probability of paternity (POP), a conditional probability of knowing whether an alleged father is the biological father of a child, was calculated using the following equation: CPI x 0.5/[CPI x 0.5 + (1- 0.5)], the CPI is the combined paternity index and 0.5 is the prior probability [15].

### Ethics approval and consent to participate

This study was approved by the Institutional Ethics Committee of CERBA/LABIOGENE and The Tribunal de Grande Instance de Ouagadougou (Deliberation N°2019-19/III-015) and conducted according to the Declaration of Helsinki. Also, written informed consent was obtained before blood collection.

## Results

### Inclusion and exclusion by the ABO/rhesus system and hemoglobin electrophoresis

Of the 14 trios, the ABO/rhesus system showed only one case of exclusion while Hb electrophoresis reported two cases of exclusion. The trio affected by the exclusion revealed by the ABO/rhesus system is different from the other two found by Hb electrophoresis. Two cases were considered inconclusive because of fetal hemoglobin (Hb F) immaturity in children (Table 2).

**Table 2:** Inclusion and exclusion results according to the ABO-rhesus/hemoglobin electrophoresis

Trio	ABO/rhesus (hemoglobin electrophoresis)			Inclusion and exclusion			Conclusion
	Mother	Child	Alleged father	ABO	Rhesus	Hemoglobin electrophoresis	
1	B+ (AC)	O+ (AA)	A+ (AA)	Inclusion	Inclusion	Inclusion	Probable inclusion
2	A+ (AA)	AB+ (AA)	B+ (AA)	Inclusion	Inclusion	Inclusion	Probable inclusion
3	B+ (AC)	B+ (AC)	B- (AA)	Inclusion	Inclusion	Inclusion	Probable inclusion
4	O+ (AA)	B+ (AF)	A+ (AA)	**Exclusion**	Inclusion	Inconclusive	**Exclusion**
5	O+ (AA)	B+ (AF)	B+ (AS)	Inclusion	Inclusion	Inconclusive	Inconclusive
6	B+ (AC)	B+ (AA)	B+ (AS)	Inclusion	Inclusion	Inclusion	Probable inclusion
7	B+ (AC)	B+ (AA)	B+ (AA)	Inclusion	Inclusion	Inclusion	Probable inclusion
8	O+ (AA)	B+ (AA)	AB+ (AA)	Inclusion	Inclusion	Inclusion	Probable inclusion
9	O+ (AA)	B+ (AC)	AB+ (AA)	Inclusion	Inclusion	**Exclusion**	**Exclusion**
10	O+ (AA)	A+ (AC)	AB+ (AA)	Inclusion	Inclusion	**Exclusion**	**Exclusion**
11	B+ (AS)	AB+ (AA)	A+ (AS)	Inclusion	Inclusion	Inclusion	Probable inclusion
12	A+ (AA)	O+ (AA)	A+ (AS)	Inclusion	Inclusion	Inclusion	Probable inclusion
13	A+ (AA)	A+ (AA)	AB+ (AC)	Inclusion	Inclusion	Inclusion	Probable inclusion
14	A+ (AA)	A+ (AA)	A+ (AA)	Inclusion	Inclusion	Inclusion	Probable inclusion

### Inclusion and exclusion according to the STR analysis

**Fig. 1:**
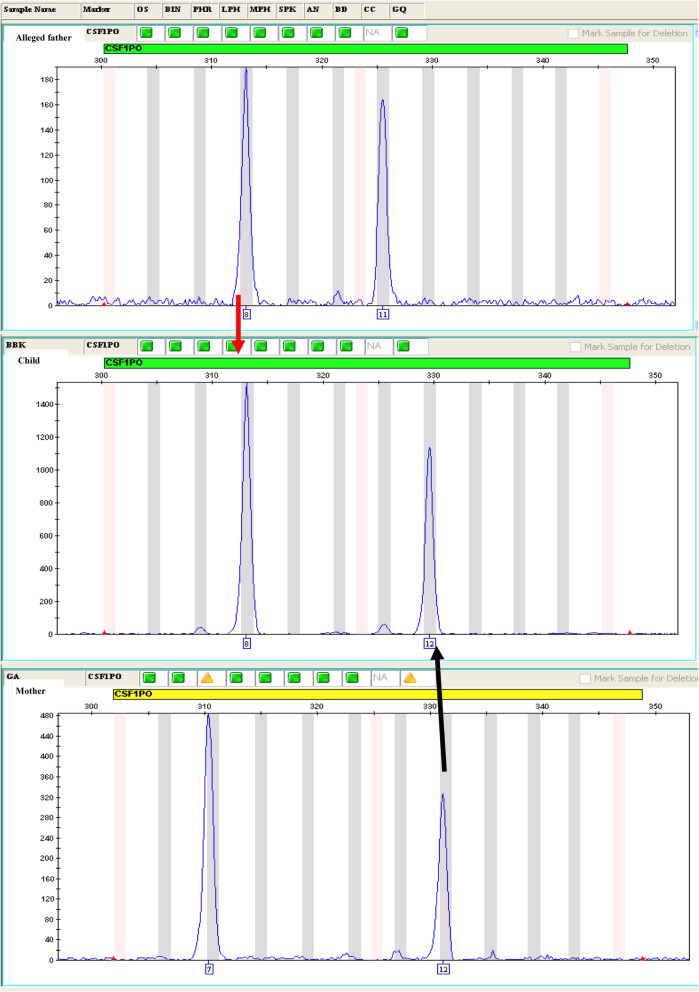
Inclusion of paternity for the trio 13: example of allele correspondence (allele 8) between alleged father and child for the locus CSF1PO

**Fig. 2:**
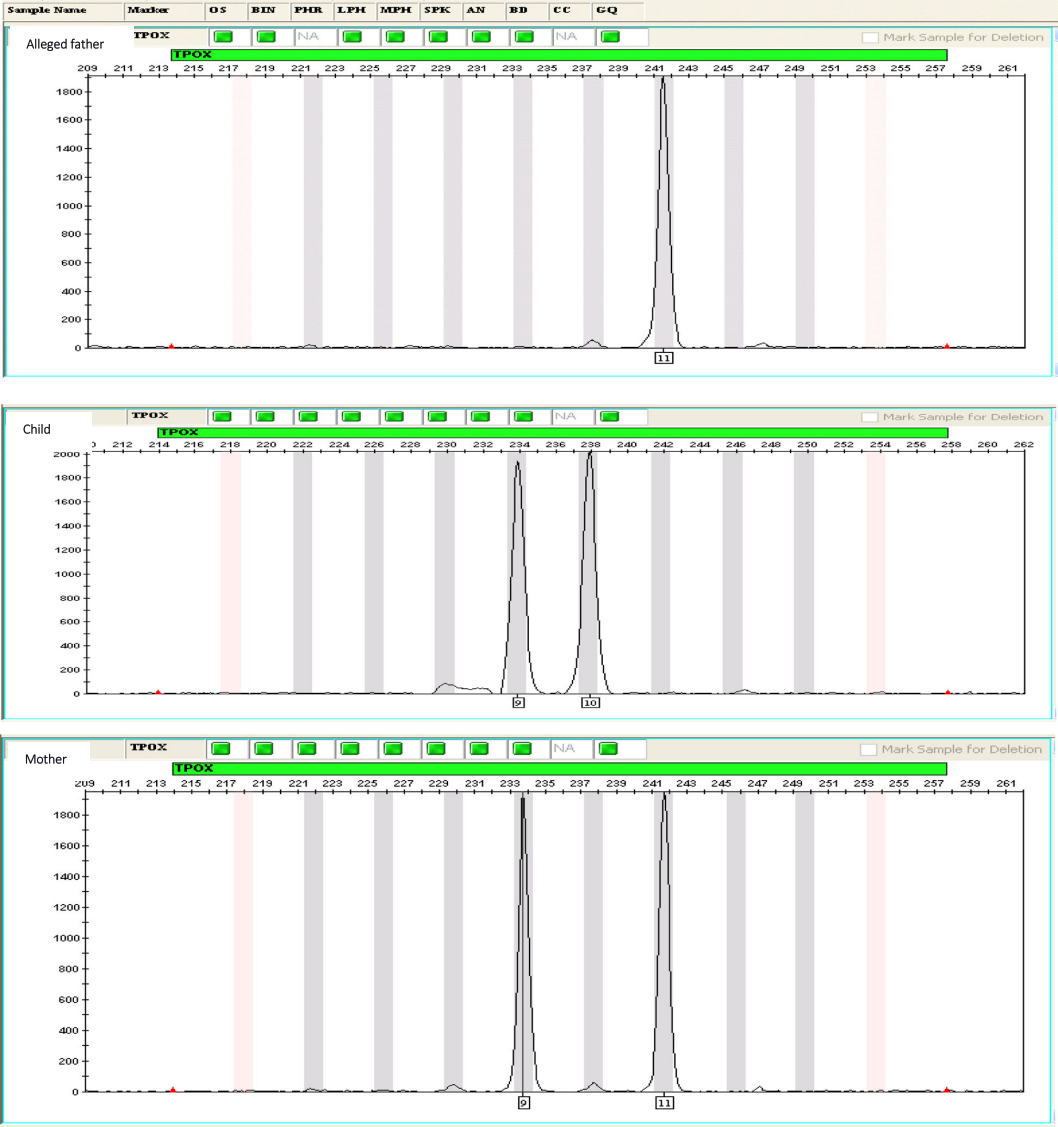
Exclusion of paternity for the trio 2: example of allele no correspondence between alleged father and child for the locus TPOX

The analysis of the 16 STRs identified the DNA profile of each trio (mother-child-alleged father). These results revealed cases of inclusion and exclusion by comparing the child’s alleles with those of both parents and by calculating the PI and POP. Of the 14 trios, 5 alleged fathers (trios 1, 3, 7, 8, and 13) were included in paternity while 9 (trios 2, 4, 5, 6, 9, 10, 11, 12, and 14) were excluded from paternity. The paternity index ranged from 0 to 37, 072, 170, and 900 and the highest POP was 99.99999999997% found in trio 3 (Table 3). Figures 1 and 2 show examples of inclusion and exclusion of paternity.

**Table 3:** Results of the combined paternity index (CPI) and the probability of paternity (POP) in trio cases

N°	Cases	CPI	POP	Conclusion of paternity
1	Trio	3, 263, 198, 110	0.99999999968	**Inclusion**
2	Trio	0	0.00	Exclusion
3	Trio	37, 072, 170, 900	0.99999999997	**Inclusion**
4	Trio	0	0.00	Exclusion
5	Trio	0	0.00	Exclusion
6	Trio	0	0.00	Exclusion
7	Trio	196, 349, 727	0.99999999490	**Inclusion**
8	Trio	12, 695, 452, 599	0.99999999992	**Inclusion**
9	Trio	0	0.00	Exclusion
10	Trio	0	0.00	Exclusion
11	Trio	0	0.00	Exclusion
12	Trio	0	0.00	Exclusion
13	Trio	2, 526, 793	0.99999996	**Inclusion**
14	Trio	0	0.00	Exclusion

Legend: *CPI* combined paternity index, *POP* probability of paternity

### Evaluation of paternity inclusion and exclusion according to the test used

Comparison of the results using the two methods revealed that 5 alleged fathers (35.71%) were included of paternity with the analysis of STR as opposed to 10 inclusions (71.43%) of paternity found with the ABO-rhesus/Hb electrophoresis method. But all 5 inclusions reported by analysis of genetic polymorphism of DNA were also found by the ABO-rhesus/Hb electrophoresis method (Table 4). Table 5 compares the inclusion/exclusion results by methods used for each trio case. Eight out of nine exclusions found with STR assay were included using ABO genotyping. Moreover, 5 inclusions (trios 2, 11, 12, 14) found with Hb electrophoresis were excluded using STR assay.

**Table 4:** Inclusion and exclusion with the ABO-rhesus/hemoglobin electrophoresis and analysis of STR

Paternity	ABO-rhesus/Hb electrophoresis	Analysis of STR
Inclusion	10 (71.43%)	5 (35.71%)
Exclusion	3 (21.43%)	9 (64.29%)
Inconclusive	1 (7.14%)	0 (0.0%)

Legend: *STR* Short Tandem Repeat, *Hb* hemoglobin

**Table 5:** Comparison of Inclusion and exclusion results by method

Trio	ABO	Rhesus	Hemoglobin electrophoresis	STR assay
1	Inclusion	Inclusion	Inclusion	Inclusion
2	Inclusion	Inclusion	Inclusion	**Exclusion**
3	Inclusion	Inclusion	Inclusion	Inclusion
4	**Exclusion**	Inclusion	Inconclusive	**Exclusion**
5	Inclusion	Inclusion	Inconclusive	**Exclusion**
6	Inclusion	Inclusion	Inclusion	**Exclusion**
7	Inclusion	Inclusion	Inclusion	Inclusion
8	Inclusion	Inclusion	Inclusion	Inclusion
9	Inclusion	Inclusion	**Exclusion**	**Exclusion**
10	Inclusion	Inclusion	**Exclusion**	**Exclusion**
11	Inclusion	Inclusion	Inclusion	**Exclusion**
12	Inclusion	Inclusion	Inclusion	**Exclusion**
13	Inclusion	Inclusion	Inclusion	Inclusion
14	Inclusion	Inclusion	Inclusion	**Exclusion**

## Discussion

To our knowledge, this study is considered the first study in Burkina Faso concerned with the disputed paternity presumption. Here, in addition to the ABO/rhesus hemoglobin electrophoresis method, we used STR assay for the identification of biological father in disputed paternity cases. The determination of paternity based on blood grouping, the rhesus factor combining Hb electrophoresis, had identified some limitations related to the profile of Hb in young infants in trios 4 and 5. This could be explained by the fact that there is still a significant proportion of fetal hemoglobin (Hb F) in infants due to their very young age [16]. Considering the blood grouping of parents and children, a match discrepancy was observed (trio4). Hemoglobin electrophoresis also showed match discrepancies in trios 9 and 10 because the Hb of the child (AC) was different from those of the mother (AA) and the presumed father (AA). However, the presumed fathers of these trios were included by ABO/rhesus. A child inherits one copy of Hb from the mother and another from the biological father [17]. Based on this principle, the two presumed fathers (9 and 10) were automatically excluded from paternity. So, the Hb electrophoresis technique had the benefit to identify exclusion cases not detected by the ABO-Rh method. The other benefit of this technique is the possibility to diagnose hemoglobinopathies cases [18–20]. These match discrepancies would make it difficult for the ABO-rhesus and Hb electrophoresis association to determine paternity. In general, the high frequencies of the ABO system alleles would make it difficult to include the presumed father in a paternity case, but could rather exclude him if, because of his blood type, he did not present the possibility of being the father. From the above, the ABO-rhesus technique associated with Hb electrophoresis used to determine paternity had limitations. The analysis of STRs consisted of determining the genetic markers in the DNA of each trio to compare the alleles of the alleged fathers with those of the children. The Identifiler Direct Kit made it possible to compare 15 alleles between the individuals in each trio. The genetic analysis of the fourteen paternity search trios comprising mother, child, and alleged father revealed 64.29% of cases of exclusion compared to 21.43% with ABO-rhesus associated with Hb electrophoresis. This trend was consistent with the studies conducted by Souiden et al., 2007 [15]. The determination of STRs would correct inclusion and exclusion errors induced by the ABO-rhesus technique associated with Hb electrophoresis. The ABO-rhesus system is easily suited to the search for the exclusion of paternity. For example, in trio 4, the mother had group O+, the child had group B+ and the alleged father had group A+. In this case, paternity was excluded with certainty and without recourse to other systems to confirm the result. Similarly, the rhesus system alone could reveal an exclusion, as is the case for example for an O- mother, O+ child, and O- father [16]. But in some cases, such as mother O+, child A1, father O+, and if the ABO system is the only exclusion system, paternity is excluded only if it can be shown that one of the parents does not have the Bombay phenotype [21]. On the other hand, the immaturity of antigens in all newborns should be considered; for example, in the following example: mother O, child A2, father A1B, the child’s blood type should be checked a few months later before reporting an exclusion, as this could be a delay in the development of the A1 antigen. For example, in the case of trios 4 and 5, the determination of paternity by Hb electrophoresis was uncertain because children had fetal hemoglobin. In addition, the Hb electrophoresis technique cannot affirm with certainty the biological father because there can be a match of the Hb when comparing the Hb of the trios (mother-child-father) without the presumed father being the biological father. Taking all these limitations into account, in the context of a paternity search, it is necessary to combine several systems (ABO, rhesus, HLA, MNS, Kell, serum systems...) [4, 22] or to use other systems such as STR genetic analysis [7]. In this study, STRs solved all the cases studied, as it was based on DNA polymorphism analysis for the identification of an individual. Based on the Bayesian probability law, we determined the PI and the CPI. These PIs maked it possible to determine the probability of paternity of an alleged father “to be” or “not to be” the biological father of a child. The results of this study showed a CPI of more than 100 million, unlike a study conducted in Egypt which found a CPI of more than 1 million [23]. The CPI can be high depending on whether the PI calculated from the allele frequencies is high or low. In the ABO system, three alleles are possible and therefore six possible genotypes are present in the human population. In contrast, STR multiplex markers produce a greater number of possible genotypes, as many alleles are present for each STR locus. Thus, although the ABO-rhesus/Hb electrophoresis is useful for excluding a person from paternity, this technique cannot be used to declare a truth inclusion of paternity. The conclusion of paternity from this technique is “Exclusion” or “probable inclusion.” The term “probable inclusion” means that there is a possibility of inclusion of paternity, but this situation needs reliable techniques to confirm. In paternity tests, the results of the probability of filiation would be either 0% to exclude someone in situations of paternity, siblings, etc., as the biological parent of a child or the same filiation or at least 99% to confirm someone as the biological parent. Legally, a 99% or greater probability of a biological relationship is considered proof of paternity [23, 24]. The inclusion of paternity comes from the fact that one of the child’s alleles is identical to one of the alleles of the alleged father for all the markers studied. While the exclusion is explained by the fact that the child did not receive any allele from the alleged father for one or more STR markers. Our results with STR assays were similar to those conducted in Egypt where STR assay was used [23]. In Poland, analysis of results obtained between 1966 and 2014 from paternity testing revealed a percentage of exclusions of 31% using serological tests (ABO, MNSs, Rh factors, Kell, Duffy, Kidd, white blood cells (HLA), serum proteins), 18% using RFLP tests and 20% using STR assay [24]. Another study in Germany using ABO genotyping by PCR-SSP and ABO grouping revealed that in 60 paternity trios with confirmed paternity of the alleged father based on STR analysis both paternity likelihood and power of exclusion of the ABO genotype was significantly higher than of the ABO phenotype. In 12 of 35 exclusion cases (34.3%) the ABO genotype also excluded the alleged father, whereas the ABO phenotype excluded the alleged father only in 7 cases (20%) [25]. As demonstrated in this study, the results of paternity tests were better when using STR analysis compared to serological tests. Every individual in the world can be identified at the molecular level based on an extremely greater level of polymorphism in the sequence of DNA which is inherited from biological parents and is identical in every cell of the body [26, 27]. In inclusion cases, the child shares the length of each STR loci with his parents because each biological parent shares 23 chromosomes for their child and in exclusion cases, the child’s length of STR loci differs between the father and mother [28]. In this study, genetic amplification of STRs excluded 9 presumed fathers and identified 5 others as the biological fathers of the children. STR markers provide sufficient discriminatory power to exclude or include an alleged father in contested paternity cases. Unlike ABO-rhesus/Hb electrophoresis techniques which are based on the agglutination of red blood cells and hemoglobin, the STR technique determines the genetic profile of the DNA which contains the genetic information that is unique to each individual [29]. Because of the limitations of ABO-Rhesus/Hb electrophoresis, the PCR technique became the standard process for DNA paternity testing because PCR technology allows amplifying a very small quantity of DNA to increase the amount of DNA up to billions of copies of the same DNA for testing and analysis [28, 30]. Furthermore, STR assays can be used to establish genetic affinity between populations [31]. In this study, the allele frequencies of the African American population provided in the kit’s user manual were used to calculate the paternity index because there are no great genetic variations between this population and West Africans [32]. Some studies were done in African countries to determine alleles frequencies [33, 34]. Moreover, the STR analysis can be used in forensic investigations. Additionally, it would be more appropriate to carry out a study to determine the allelic frequencies of the 16 STRs specific to the population of Burkina Faso.

## Conclusion

In Burkina Faso, ABO-rhesus/hemoglobin electrophoresis tests have long been used for paternity exclusion. Because of the limitations of these conventional tests, STR analysis has become the reference technique not only for parentage testing but also for forensic analysis.

## Data Availability

Not applicable

## Abbreviations

CPI: Combined paternity index
DNA: Deoxyribonucleic
HbF: Fetal hemoglobin
PCR: Polymerase chain reaction
PI: Paternity index
POP: Probability of paternity
POP4: Performance-optimized polymer 4
STR: Short tandem repeat
